# Synthesis and Characterization of Aluminum 2-Carboxyethyl-Phenyl-Phosphinate and Its Flame-Retardant Application in Polyester

**DOI:** 10.3390/polym11121969

**Published:** 2019-11-29

**Authors:** Zhongying Yao, Xinxin Liu, Lijun Qian, Yajun Chen, Bo Xu, Yong Qiu

**Affiliations:** 1 School of Materials Science and Mechanical Engineering, Beijing Technology and Business University, Beijing 100048, China; yzybtbu@163.com (Z.Y.); chenyajun@th.btbu.edu.cn (Y.C.); xubo@btbu.edu.cn (B.X.); yongqiu@btbu.edu.cn (Y.Q.); 2 Engineering Laboratory of Non-Halogen Flame Retardants for Polymers, Beijing 100048, China; 3 National-Certified Enterprise Technology Center, Kingfa Science and Technology Co., Ltd., Guangzhou 510520, China; lxxbtbu@163.com

**Keywords:** polyester textile, flame retardant, coating, phosphinate

## Abstract

A flame retardant aluminum 2-carboxyethyl-phenyl-phosphinate (CPA-Al) was synthesized through the salification reaction. The molecular structure of CPA-Al and thermal stability were characterized by solid nuclear magnetic resonance, Fourier transform infrared spectroscopy, and thermogravimetric analysis. Subsequently, CPA-Al mixed in polyurethane was coated on polyester textile to obtain flame-retardant samples. The addition of 14.7 wt.% CPA-Al in textile sample can bring a limited oxygen index (LOI) value of 24.5%, 0 s after flame time, and the vertical burning B1 rating. Meanwhile, the incorporated CPA-Al reduced the peak heat release rate, total heat release, average effective heat of combustion, and increased the charring capacity of polyester textiles in contrast to the samples without CPA-Al. CPA-Al exerted not only its flame inhibition effect in gas phase, but also the charring and barrier effect in the condensed phase. Besides, with an increasing CPA-Al ratio in polyester textile, the contact angle gradually decreased from 123.6° to 75.6°, indicating that the surficial property of coating from hydrophobic to hydrophilic, thereby increasing the moisture permeability of polyester textile.

## 1. Introduction

In the past several decades, polyester textile has been widely applied to garment, upholstery, and decoration, etc. [1,2], because of its excellent properties, such as low cost, being resistant to chemicals, dimensional stability, and good physical features [3,4,5,6,7,8]. However, polyester textile is flammable and it will melt dripping during combustion, which caused potential fire risks to people’s lives and property [9,10]. Therefore, it is necessary to impose flame retardancy to textile in work clothes and tents applications [11,12].

In recent years, some researchers have been trying several kinds of flame retardants, such as brominated flame retardants [13], phosphorus-containing flame retardant [14,15], intumescent flame retardant [16,17], and nanometer flame retardant [18,19,20] to improve the flame retardancy of polyester textile. Some researchers have been trying to combine some known efficient phosphorus-based characteristic structures or functional groups, such as phosphonate and phosphinate [21,22,23,24], cyclotriphosphazene [25,26,27,28], and phosphinate-POSS [29,30] to prepare the flame-retardant polyester textile. 

There are three ways to acquire flame retardancy to polyester textile [31]: copolymerizing flame-retardant monomer in polyester [32]; incorporating flame-retardant additive into polyester during processing [33]; and treating polyester textile in flame-retardant solution [34]. Treating polyester textile is a feasible method for obtaining flame-retardant textile due to its easy processing way. The treating methods include photo-induced grafting reactions [35], oxygen plasma treatment [36], layer-by-layer (LbL) assembly [37,38,39], dyeing-like process [40], or sol-gel process [3]; traditional coatings by means of spray-, brush-, or roller techniques [41] and so on. More importantly, a simple and efficient treatment method can reduce the production cost and greatly save manual labour and material resources.

In this thesis, a kind of phosphinate, which was named aluminum 2-carboxyethyl-phenyl-phosphinate (CPA-Al), was successfully synthesized and characterized. Afterwards, CPA-Al incorporated into polyurethane (PU) as back coating materials to prepare flame-retardant polyester textile, which was a simple and convenient way. The flame-retardant properties, mechanism, and surficial property of CPA-Al on polyester textile were investigated. 

## 2. Experimental

### 2.1. Materials 

3-Hydroxyphenylphosphinyl-propanoic acid (CPA) was provided by Dezhou Changxing Chemical New Materials Research and Development Co., Ltd. (Dezhou, China). Aluminum chloride hexahydrate was purchased from Sinopharm Chemical Reagent Co. Ltd. (Beijing, China). Polyester textile (T, weighting 155 g/m^2^, thickness 0.20 mm) was supplied by Jiangsu Shixing Textile Co., Ltd. (Jiangsu, China). Toluene was supplied by Beijing Chemical Works (Beijing, China). Polyurethane (PU, HKW-3055), which was supplied by Haina Environmental Science and Technology (Shandong, China), was prepared by polyester polyols (2024, prepared by adipic acid, ethylene glycol and 1, 4-butylene glycol) that were reacting with diphenyl methane di-isocyanate (MDI) and mixing *N*,*N*-dimethylformamide (DMF) (70 wt % in PU).

### 2.2. Synthesis of CPA-Al

**Scheme 1:**
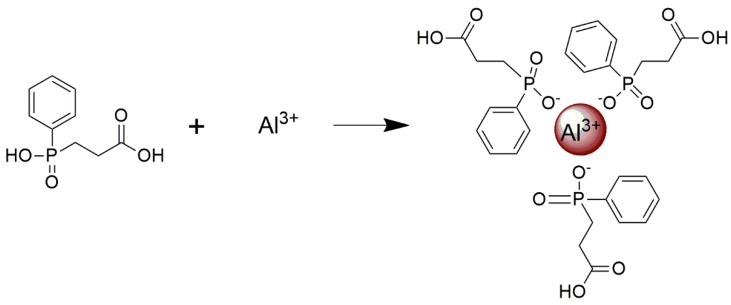
Synthesis route of 2-carboxyethyl-phenyl-phosphinate (CPA-Al).

CPA (128.4 g, 0.6 mol) and 400 mL water were fed into a 1000 mL three-neck flask with a mechanical stirrer. The mixture was stirred and heated to 70 °C in water bath until the CPA completely dissolved in deionized water. Subsequently, the reaction temperature was increased to 90 °C and then stabilized for 30 min. A solution of aluminum chloride hexahydrate (96.6 g, 0.4 mol) in 200 mL water was dripped into the mixture within 30 min. While the solution of aluminum chloride hexahydrate was added into the reaction system, the mixture turned to white emulsion and the reaction was carried out for another 3 h. The reaction mixture was filtered to obtain the solid product and the product was then washed and filtered with water at 80 °C until the PH value of the filtrate remained stable. Finally, the product was dried in a vacuum oven at 120 °C for 3 h. Scheme 1 shows the reaction formula. The yield of CPA-Al was up to 90%. 

### 2.3. Preparation of Flame-Retardant Textiles and the Control Samples

PU, toluene, and CPA-Al were stirred and completely blended at room temperature in a beaker to prepare PU coating. Table 1 lists the formulas of PU, toluene, and CPA-Al in each sample.

The polyester textile was pulled and fixed on the holder and PU coating was then carried out on the back of textiles under about 60 N pressure. The coated textiles were then placed in a drying oven at 160 °C for 3 min. The flame-retardant textiles were labeled as 7.1%CPA-Al/PU/T, 10.3%CPA-Al/PU/T, and 14.7%CPA-Al/PU/T based on the mass ratio of CPA-Al in each sample. The control sample PU/T was also prepared in the same manner, but without the addition of the flame retardant CPA-Al. Table 1 lists the mass fraction of CPA-Al in each textile sample. 

### 2.4. Characterization

FTIR spectra were obtained while using a Nicolet iN10MX-type spectrometer (Thermo Nicolet Corp., Madison, WI, USA) over the frequency range of 500 to 4000 cm^−1^. Thirty-two scans and eight cm^−1^ spectral resolutions were used for each measurement. The powdered samples were thoroughly mixed with KBr and then pressed into pellets. 

The ^1^H nuclear magnetic resonance (^1^H NMR), ^13^C NMR, ^27^Al NMR, and ^31^P NMR data were obtained while using a Bruker 400 MHz WB Solid-State NMR Spectrometer (Karlsruhe, Germany).

Thermogravimetric analysis (TGA) was performed using a PerkinElmer STA 8000 thermal gravimetric analyzer (Perkin Elmer, Waltham, MA. USA). The sample was placed in a platinum crucible and then heated from 50 to 700 °C at the rate of 20 °C/min. in N_2_ atmosphere. All of the tests were repeated three times, and the typical TGA data were reproducible within ±5%. 

The limited oxygen index (LOI) value was measured while using an FTT (Fire Testing Technology, London, UK) Dynisco LOI instrument according to ASTM D2863-97 (sample dimension: 140.0 mm × 52.0 mm). The LOI measurement for each specimen was repeated three times, and the error values were ±0.3%. The vertical burning test (Fire Testing Technology, London, UK) was performed while using an FTT0082 instrument according to GB/T 5455 (sample dimension: 300.0 mm × 80.0 mm). The cone calorimeter test (Fire Testing Technology, London, UK) was performed while using an FTT0007 cone calorimeter according to ISO5660 at an external heat flux of 50 kW/m^2^ (sample dimension: 100.0 mm × 100.0 mm). The measurement for each specimen was repeated twice, and the error values of the typical cone calorimeter data were reproducible within ±5%.

A STA 8000 TGA thermogravimetric analyzer (Perkin Elmer, Waltham, MA. USA) was coupled to Frontier infrared spectrometer (Perkin Elmer, Waltham, MA. USA) (TGA-FTIR) to detect volatile pyrolysis products. Each sample was placed in an alumina crucible and then heated from 50 to 700 °C at a heating rate of 20 °C/min. under N_2_ atmosphere. The thermogravimetric analyzer and FTIR spectrometer were connected by pipeline at 280 °C.

The SEM images of the residues after cone calorimeter test were obtained while using a Phenom Pro Desktop scanning electron microscope (Phenom World, Eindhoven, Netherlands) at high vacuum conditions with a voltage of 5 kV. The tested specimens were taken from the outer layer of carbon residues after the cone calorimeter test.

The element contents of residues from cone calorimeter were investigated via a PerkinElmer PHI 5300 ESCA X-ray photoelectron spectrometer (XPS, Waltham, MA, USA). The tested specimens were obtained from the surface of residues with sufficiently mixed and grinded residues, and the results were the average of the three times repeated tests, which were all reproducible within ±5%.

The contact angles of different polyesters were measured with a Dataphysics OCA-35 contact angle analyzer (DataPhysics Instruments GmbH, Filderstadt, Germany) at room temperature at an ambient atmosphere.

The water vapor permeability of polyesters samples was tested with a relative humidity (RH) of 90% and at the temperature of 23 °C, according to Standard GB/T 1037-1988 (circular samples dimension: 100.0 mm diameter, 0.2 mm thickness). The results were the average of the three times repeated tests. 

## 3. Results and Discussion

### 3.1. Characterization of the Structure and Thermal Stability of CPA-Al

**Figure 1:**
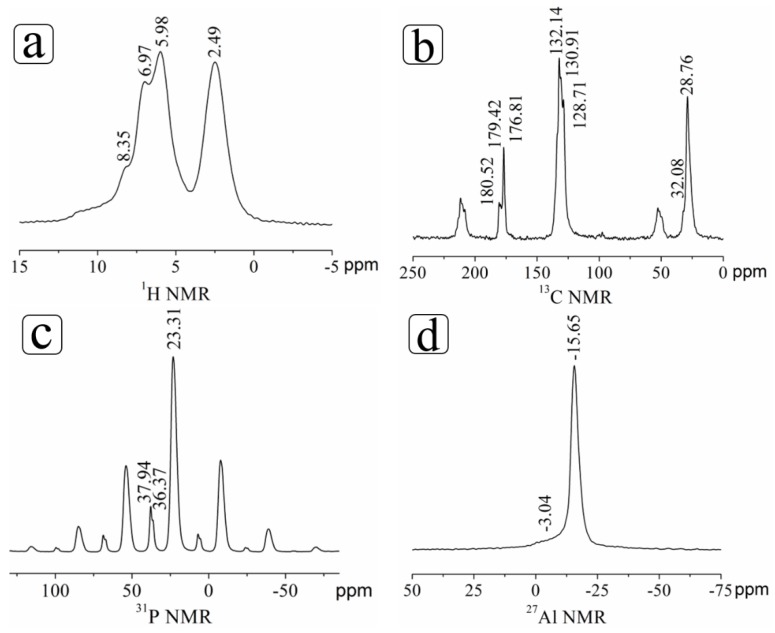
Solid-state NMR spectra of CPA-Al. (**a**) ^1^H NMR; (**b**) ^13^C NMR; (**c**) ^31^P NMR; and (**d**) ^27^Al NMR of CPA-Al.

**Figure 2:**
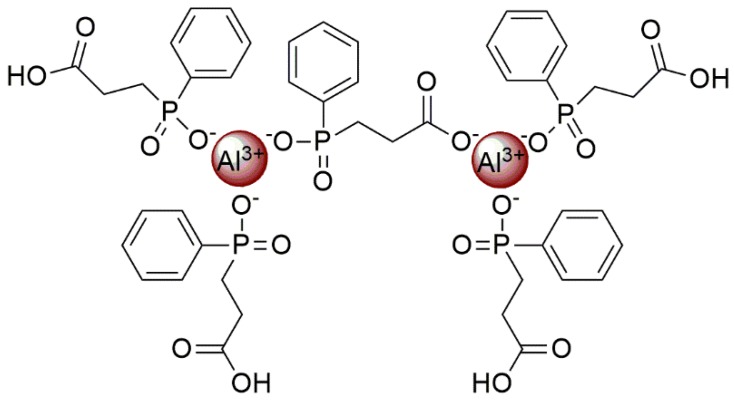
The chemical structure of CPA-Al main by-product.

^1^H solid-state NMR, ^13^C solid-state NMR, ^31^P solid-state NMR, and ^27^Al solid-state NMR confirm the structure of CPA-Al, as in Figure 1. In Figure 1a, the chemical shift at 2.49 ppm was attributed to H atoms in ethyl group. H atoms in the benzene ring caused the chemical shift at 5.98, 6.97, and 8.35 ppm. The H atoms in carboxyl should cause the chemical shift between 10–12 ppm. In Figure 1b, the chemical shift at 28.76 and 32.08 ppm was assigned to C atoms in –CH_2_–CH_2_ group. The chemical shift between 128.71–135.00 ppm was caused by C atoms in the benzene ring. The chemical shift at 176.81–180.52 ppm was attributed to C atoms in carboxyethyl group. There was not only one chemical shift between 176.81–180.52 ppm, so the reaction might be generated some by-products. The chemical shift at 23.31 ppm in ^31^P NMR was caused by the C–P–C. Combining the results of ^1^H NMR, ^13^C NMR, and ^31^P NMR, a little of –COOH should react with Al^3+^, and Figure 2 shows the structure of the by-product. Therefore, the shift at 36.27–37.94 was caused by the C–P–C bond, which was affected by the –COO– of by-product. In Figure 1d, the strong peak at −15.65 ppm was assigned to aluminum atoms in hypophosphite. The aluminum atoms from aluminum carboxylate structure in by-product caused the weak peak at −3.04 ppm. The integral area of the two peaks shows that the ratio of Al in by-product is very rare.

**Figure 3:**
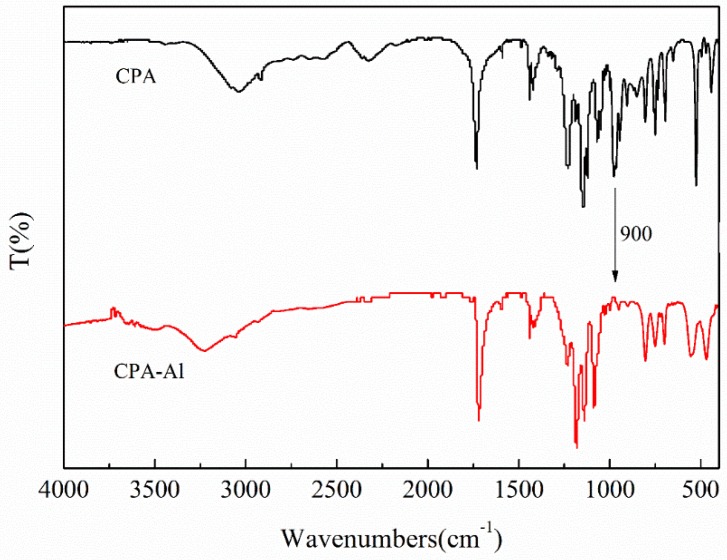
FTTR spectra of CPA-Al and 3-Hydroxyphenylphosphinyl-propanoic acid (CPA).

Figure 3 shows the FTTR spectra of reactant CPA and product CPA-Al. The spectra exhibit that the absorption band at 900 cm^−1^ in CPA spectrum disappeared in CPA-Al. The absorption band at 900 cm^−1^ represents the deformation vibration of out of plane O–H. The disappearance of this band implies that O–H bond transformed to O^−^Al^3+^ structure. Absorption at 1721 cm^−1^ (C=O) has no obvious change in both CPA and CPA-Al, indicating that –COOH hardly did attend the salification reaction. Combining with previous discussion in solid-state NMR, it can be deduced that only a little –COOH group participated in the salification reaction and little by-products were formed. CPA-Al was successfully synthesized, according to the results. 

**Figure 4:**
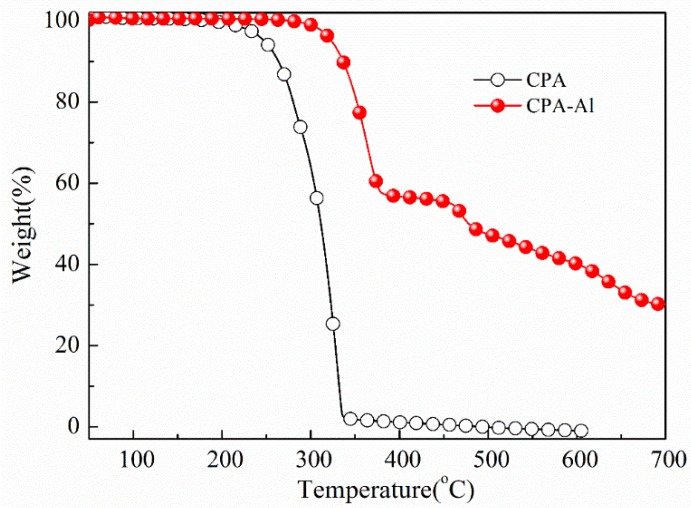
TGA curves of CPA-Al and CPA.

The thermal stability of CPA-Al was also tested by TGA, and Figure 4 and Table 2 show the results. The thermal stability of CPA-Al was outstandingly enhanced when compared with raw material CPA. The onset degradation temperature (T_d, 1%_) of CPA-Al reached up to 300 °C. CPA at 600 °C decomposed completely and no residue nearly was reserved. However, the residue yield of CPA-Al at 600 °C was 40.1%. All of these results confirm that CPA-Al possessed higher thermal stability and better charring ability, which contributes to bringing better barrier effect to materials.

### 3.2. LOI and Vertical Burning Test.

CPA-Al was applied in PU coating of polyester textile to investigate its flame retardancy. The flame-retardant properties of the polyester textiles were evaluated while using LOI and vertical burning tests. Table 3 presents the corresponding results. The LOI values of PU/T were only 20.0%. After the CPA-Al was incorporated into the polyester coating, the LOI values of samples gradually increased. The LOI value increased to 24.5% when the mass fraction of CPA-Al in sample was 14.7 wt.%.

The after flame time after 12 s exposure time under fire of vertical burning test for PU/T was 32.5 s until the textiles were totally burned out. After flame-retardant CPA-Al was incorporated into the PU, the after flame time shortened obviously when compared with PU/T. It is also clearly observed that the burning distances decreased with increasing mass fraction of CPA-Al in PU coatings. When the mass fraction of CPA-Al in samples reached 14.7% (14.7%CPA-Al/PU/T), it had not any dripping observed and the rating reached to B1 with the 0 s after flame time during the vertical burning test, which disclosed the anti-dropping effect of CPA-Al on polyester textiles during combustion.

**Figure 5:**
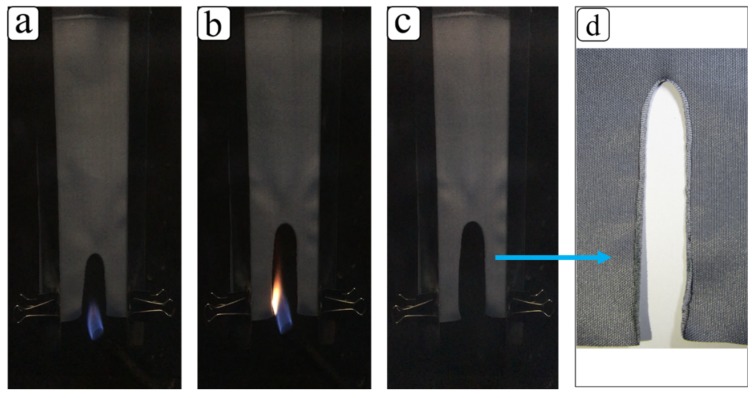
Digital photos of 14.7%CPA-Al/PU/T during combustion of vertical burning test. (**a**) 5 s; (**b**) 10 s; (**c**) 15 s; and (**d**) Partial digital photo.

Figure 5 was the digital photos of 14.7%CPA-Al/PU/T at different points during the combustion of vertical burning test. The polyester textiles were not ignited by fire and the damage length of the 14.7%CPA-Al/PU/T was caused by the heat of flame and not by combustion. The edge of damaged polyester textile was curly after firing. There was no obvious charring behavior around the damage edge of 14.7%CPA-Al/PU/T in Figure 5d. All of the phenomena disclose that CPA-Al should effectively inhibit the free radical chain reaction in the gaseous phase during combustion.

### 3.3. Cone Calorimeter Test

**Figure 6:**
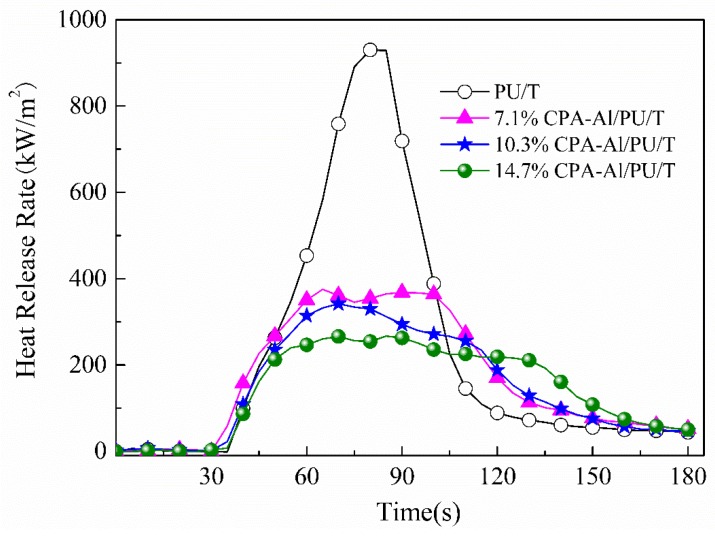
Heat release rate (HRR) curves of CPA-Al polyester textiles.

**Figure 7:**
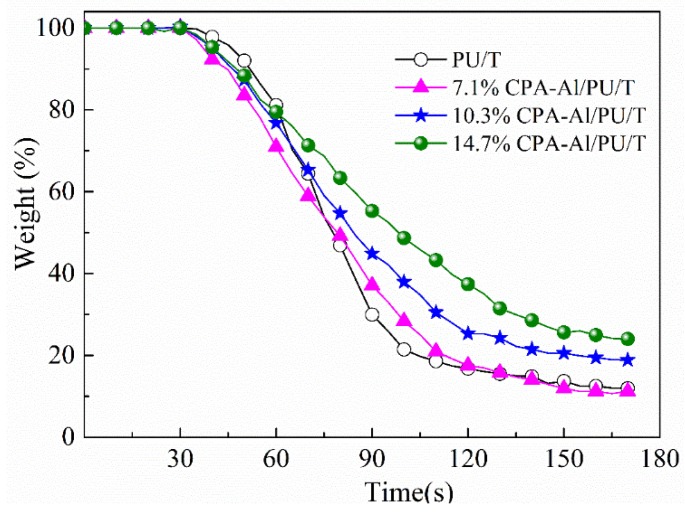
Mass loss rate (MLR) curves of CPA-Al polyester textiles.

The cone calorimeter test was conducted to sufficiently investigate the flame-retardant behaviors of CPA-Al in the polyester. Figure 6 and Figure 7 respectively show the curves of heat release rate (HRR) and the curves of mass loss rate (MLR), and Table 4 summarizes the partial typical characteristic parameters, such as peak of heat release rate (PHRR), total smoke release (TSR), average of effective heat of combustion (av-EHC), total heat release (THR), average CO yield (av-COY), average CO_2_ yield (av-CO_2_Y), and residue. These data of flame-retardant samples mentioned above were calculated from time 30 s to time 165 s and the dates of PU/T were calculated from 35 to 125 s, according to the curves of heat release rate.

As shown in Table 4 and Figure 6, the PHRR values of CPA-Al/PU/T were distinctly reduced and burning intensity was effectively inhibited. The PHRR of 14.7%CPA-Al/PU/T fell by 71.3% when compared with that of PU/T. There was a sharp peak from the start to the end in the HRR curves of PU/T but the HRR curve of CPA-Al/PU/T was gentle, indicating that CPA-Al exerted better charring and barrier effect. In Table 4, all of the av-EHC values of CPA-Al/PU/T were lower than that of PU/T, and they all gradually decreased with an increasing mass ratio of CPA-Al in samples. EHC can be used to measure burning ratio of combustible volatile gas. The reduction of the av-EHC values indicated that the incorporation of CPA-Al could effectively inhibit the combustion of volatile gas and terminate the free radical chain reaction in gaseous phase.

According to Figure 7 and Table 4, with an increasing ratio of CPA-Al in textiles, the total mass loss (TML) values at the end of combustion obviously increase and more residues were produced, which implies that CPA-Al had an excellent charring effect. The formation of more residues not only enhanced the barrier effect to fire, but also decreased the release of fuels. Due to the incorporation of CPA-Al, the decrease of fuels testified by TML and the incomplete combustion of the flammable gas determined by av-EHC values all contributed to the lower burning intensity and total heat release. The decrease of fuels testified by TML and the incomplete combustion of the flammable gas determined by av-EHC values all contributed to lower burning intensity and total heat release due to incorporation of CPA-Al. In Table 4, the THR values gradually decreased with an increasing fraction of CPA-Al in PU coating, which corresponded to the results discussed. 

Similarly, the incomplete combustion in the gaseous phase also led to the more smoke and the more av-COY, which the results in Table 4 proved. The incomplete contents in the gaseous phase would result in the more solid char particles, and accordingly the smoke density of flame-retardant samples would increase. According to Table 4, the flame-retardant samples with CPA-Al produced more CO, less CO_2_, and the higher TSR values. The addition of CPA-Al in samples obviously inhibited the combustion of volatile gas and exerted flame-retardant action in gaseous phase.

### 3.4. TGA and TGA-FTIR Analysis

**Figure 8:**
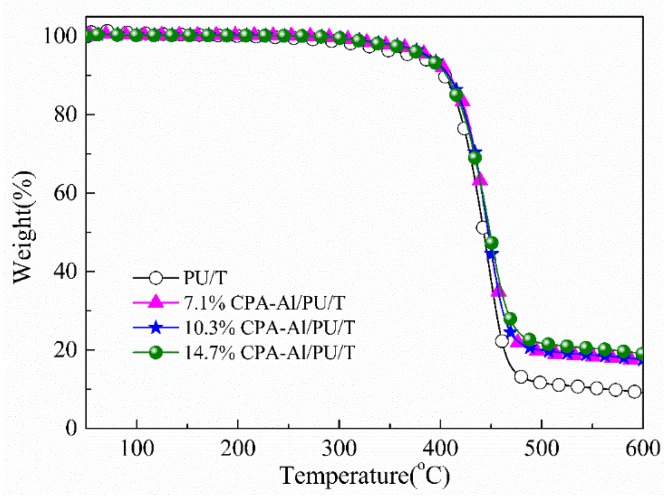
TGA curves of polyester textiles.

TGA analysis also further testifies the results in cone calorimeter. Figure 8 shows the TGA curves of polyesters, and Table 5 lists some typical dates. The residual yields at 600 °C of PU/T is only 9.2 wt %, whereas that of CPA-Al/PU/T at 600 °C is from 17.2% to 19.0% with increasing mass fraction of CPA-Al. Therefore, the result further showed the promotion action of CPA-Al on the charring yields of the matrix.

**Figure 9:**
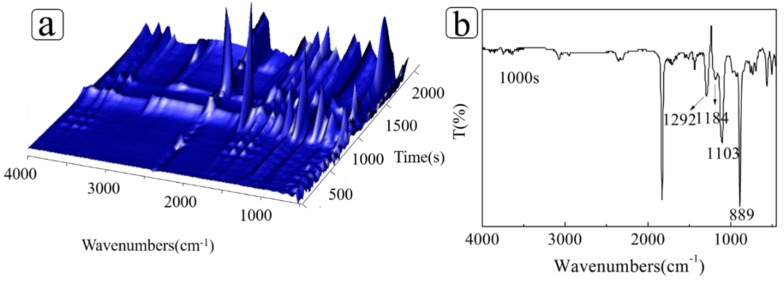
(**a**) Three-dimensional (3D) FTIR spectrum of the pyrolysis gas products of CPA-Al from TGA; (**b**) FTIR spectra of pyrolysis gas products of CPA-Al at the maximum gas release rate.

Figure 9 shows the three-dimensional (3D) FTIR spectrum of the pyrolysis gas products of CPA-Al from TGA in order to explore the flame retardant mechanism of CPA-Al in gas phase. The release rate of the decomposed rapidly increased to a maximum value at 1000 s and decreased until around 1300 s, where it had another larger value. In Figure 9a, the gas release trend was in keeping with the TGA results of CPA-Al. Figure 9b shows the FTIR spectra of pyrolysis gas products of CPA-Al at the maximum gas release rate. In this spectrum, the peaks at 1184 and 1103 cm^−1^ are attributed to PO_2_^−^ anion absorption and the peaks at 1292 and 889 cm^−1^ can be assigned to P=O and P–O bonds, respectively. The phosphorus-containing groups can quench radicals and reveal the flame-retardant effect in gas phase. The presence of PO_2_ and PO in the spectrum provides the basis for CPA-Al in gas phase of flame inhibition effect.

### 3.5. The Residue Analysis from Cone Calorimeter Test

**Figure 10:**
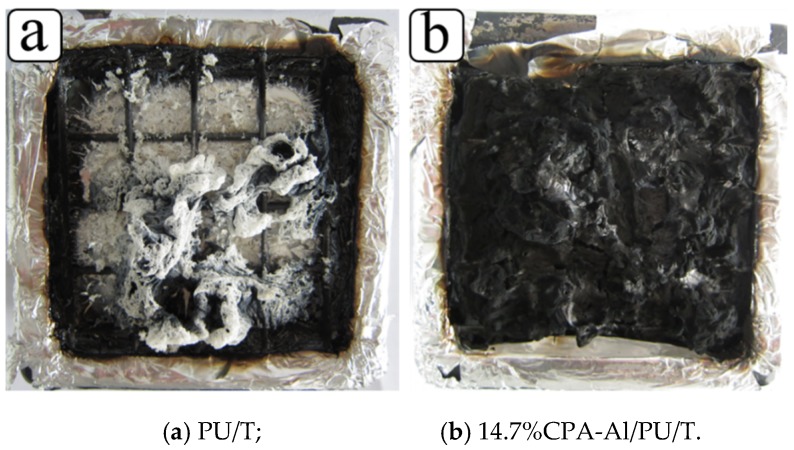
Digital photos of polyester residues from cone calorimeter.

Figure 10 shows the digital photos of the residues from cone calorimeter test. As shown in Figure 10a, the PU/T was almost completely combusted and only a few residues of PU/T were left. After CPA-Al was incorporated into PU/T, as shown in Figure 10b, the residue yields and quality of 14.7%CPA-Al/PU/T obviously increased. CPA-Al promoted the charring process of polyester textiles during combustion and exerted a barrier effect of the char layer in condensed phase.

**Figure 11:**
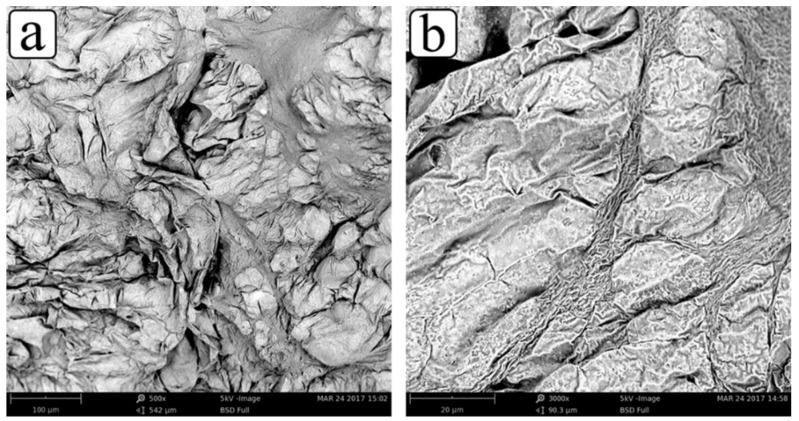
SEM photos of 14.7%CPA-Al/PU/T residue from cone calorimeter. (**a**) 500×; (**b**) 3000×.

SEM analysis of 14.7%CPA-Al/PU/T was conducted in order to explore the microscopic morphology of residue further, and the results are shown in Figure 11. There were many bosomy and sealed bubble-shaped structures with different sizes in Figure 11a. In Figure 11b, the bubble-shaped structure was clearly observed and the structure is continuous and sealed with many wrinkles. The residue structure of 14.7%CPA-Al/PU/T can inhibit the exchange of the fuel and oxygen and efficiently protect the polyester from the flame. The continuous and sealed char layer of 14.7%CPA-Al/PU/T blocked the volatile gas generated by polyester, and then cut off the fuel supply, and ultimately weakened the combustion intensity. The results confirm that the incorporation of CPA-Al should promote the formation of sealed and flexible char layer. The results also further support the flame-retardant effect of CPA-Al in the condensed phase.

The element contents of PU/T and CPA-Al/PU/T residues from the cone calorimeter tests were determined via XPS. Table 6 lists the results. The phosphorus content in residues has an obvious up tendency with increasing the mass ratio of CPA-Al in samples, indicating that more phosphorus contents joined the charring process and promoted the formation of phosphorus-rich residue, thereby bringing a better barrier effect on heat and fire.

### 3.6. Barrier Performance of CPA-Al/PU/T

**Figure 12:**
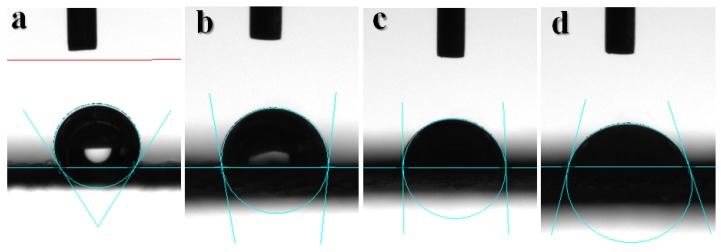
Digital images of the contact angle experiment. (**a**) PU/T; (**b**) 7.1%CPA-Al/PU/T; (**c**) 10.3%CPA-Al/PU/T; and, (**d**) 14.7%CPA-Al/PU/T.

Table 7 shows the results of water vapor permeation. The amounts water vapor permeation of CPA-Al/PU/T samples all highly increased when compared with PU/T and the water vapor permeation of 14.7%CPA-Al/PU/T was up to 1648.6 g/m^2^·day. The addition of CPA-Al improved the moisture permeability of polyester textiles. The contact angle test was carried out to probe the reason for the change of water vapor permeation, and the results are shown in Table 7 and Figure 12. With an increasing CPA-Al ratio in polyester textile, the contact angle gradually decreased from 123.6° to 75.6°. Due to the carboxyl functional group of CPA-Al being a hydrophilic group, the contact angle of polyester textile will be reduced, indicating that the polyester textile changed their surficial property from hydrophobic to hydrophilic [42,43,44]. That is why the amounts of water vapor permeation gradually increased [45].

## 4. Conclusions

A flame-retardant additive CPA-Al was successfully synthesized and characterized. The polyester textile with 14.7 wt.% CPA-Al obtained a LOI value of 24.5%, after a flame time of 0 s, self-extinguishment, shorter burning distance, and B1 rating in the vertical flame test. The incorporated CPA-Al also decreased the PHRR, av-EHC, THR, and increased the charring capacity of polyester textile. The flame-retardant effect of CPA-Al resulted from not only flame inhibition effect in gaseous phase, but also the charring and barrier effect of flexible and sealed char layer in the condensed phase. Therefore, CPA-Al in the PU coating can endow polyester textile with better flame-retardant effect. Besides, with an increasing CPA-Al ratio in polyester textile, the contact angle gradually decreased from 123.6° to 75.6°. The result revealed that CPA-Al could increase the moisture permeability of polyester textile by means of changing the surficial property of coating from hydrophobic to hydrophilic [46]. 

## Figures and Tables

**Table 1:** Formulas of the different textile samples.

Samples	Polyester Textile (g)	PU (g)	CPA-Al	
			g	wt %
PU/T	139	18	0	0
7.1%CPA-Al/PU/T	139	18	12	7.1
10.3%CPA-Al/PU/T	139	18	18	10.3
14.7%CPA-Al/PU/T	139	18	27	14.7

**Table 2:** Thermogravimetric analysis (TGA) parameters of CPA-Al and CPA.

Samples	T_d,1%_ (°C)	T_d,5%_ (°C)	Residuals at 600 °C (wt %)	Residuals at 700 °C (wt %)
CPA	214	248	0	--
CPA-Al	300	324	40.1	30.1

**Table 3:** Limited oxygen index (LOI) value and vertical burning test of textile samples.

Samples	LOI (%)	Vertical Burning Test			
		After Flame Time (s)	The Length of Combustion/cm	Dripping	Rating
PU/T	20.0	32.5	30.0	Yes	NR
7.1%CPA-Al/PU/T	23.6	30.0	16.2	Yes	NR
10.3%CPA-Al/PU/T	23.8	8.8	9.8	Yes	B2
14.7%CPA-Al/PU/T	24.5	0	7.4	No	B1

**Table 4:** Cone calorimeter data of CPA-Al polyester samples.

Samples	PHRR (kW/m^2^)	av-EHC (MJ/m^2^)	THR (MJ/m^2^)	TSR (m^2^/m^2^)	av-COY (kg/kg)	av-CO_2_Y (kg/kg)	Residue (wt %)
7.1%CPA-Al/PU/T	375	22.6	29.8	1323	0.11	2.48	11.2
10.3%CPA-Al/PU/T	342	19.4	26.6	1395	0.15	2.69	18.9
14.7%CPA-Al/PU/T	267	16.8	25.2	1438	0.14	2.20	24.0
PU/T	930	25.5	38.2	989	0.07	2.80	11.9

**Table 5:** TGA parameters of polyester textiles.

Samples	T_d, 1%_ (°C)	T_d, 5%_ (°C)	Residuals at 600 °C (wt %)
PU/T	286	375	9.2
7.1%CPA-Al/PU/T	315	388	17.2
10.3%CPA-Al/PU/T	321	388	17.5
14.7%CPA-Al/PU/T	317	384	19.0

**Table 6:** Element contents of residues from the cone calorimeter test.

Samples	Element Content (wt %)				
	C	N	O	Al	P
7.1%CPA-Al/PU/T	77.64	2.07	15.98	1.74	2.57
10.3%CPA-Al/PU/T	70.67	1.88	19.17	3.36	4.92
14.7%CPA-Al/PU/T	69.55	3.01	19.34	2.81	5.29
PU/T	75.84	2.98	21.18	--	--

**Table 7:** Water vapor permeation and contact angles of CPA-Al/PU/T.

Samples	Water Vapor Permeation (g/m^2^·day)	Contact Angle (°)
PU/T	690.3	123.6
7.1%CPA-Al/PU/T	1091.2	99.8
10.3%CPA-Al/PU/T	1280.4	92.5
14.7%CPA-Al/PU/T	1648.6	75.6
